# Genetic Dissection of Resistance to the Three Fungal Plant Pathogens *Blumeria graminis*, *Zymoseptoria tritici*, and *Pyrenophora tritici-repentis* Using a Multiparental Winter Wheat Population

**DOI:** 10.1534/g3.119.400068

**Published:** 2019-03-22

**Authors:** Melanie Stadlmeier, Lise Nistrup Jørgensen, Beatrice Corsi, James Cockram, Lorenz Hartl, Volker Mohler

**Affiliations:** * Bavarian State Research Center for Agriculture, Institute for Crop Science and Plant Breeding, Freising 85354, Germany; † Technical University of Munich, TUM School of Life Sciences Weihenstephan, Freising 85354, Germany; ‡ Aarhus University, Department of Agroecology, Slagelse 4200, Denmark, and; § John Bingham Laboratory, NIAB, Huntingdon Road, Cambridge CB3 0LE, United Kingdom

**Keywords:** QTL analysis, MAGIC, Powdery mildew, Septoria tritici blotch, Tan spot, Multiparent Advanced Generation Inter-Cross (MAGIC), multiparental populations, MPP

## Abstract

Bread wheat (*Triticum aestivum* L.) is one of the world’s most important crop species. The development of new varieties resistant to multiple pathogens is an ongoing task in wheat breeding, especially in times of increasing demand for sustainable agricultural practices. Despite this, little is known about the relations between various fungal disease resistances at the genetic level, and the possible consequences for wheat breeding strategies. As a first step to fill this gap, we analyzed the genetic relations of resistance to the three fungal diseases – powdery mildew (PM), septoria tritici blotch (STB), and tan spot (TS) – using a winter wheat multiparent advanced generation intercross population. Six, seven, and nine QTL for resistance to PM, STB, and TS, respectively, were genetically mapped. Additionally, 15 QTL were identified for the three agro-morphological traits plant height, ear emergence time, and leaf angle distribution. Our results suggest that resistance to STB and TS on chromosome 2B is conferred by the same genetic region. Furthermore, we identified two genetic regions on chromosome 1AS and 7AL, which are associated with all three diseases, but not always in a synchronal manner. Based on our results, we conclude that parallel marker-assisted breeding for resistance to the fungal diseases PM, STB, and TS appears feasible. Knowledge of the genetic co-localization of alleles with contrasting effects for different diseases, such as on chromosome 7AL, allows the trade-offs of selection of these regions to be better understood, and ultimately determined at the genic level.

Common wheat (*Triticum aestivum* L.) is the most important food crop in the European Union with a total share of near half of cereal production (Eurostat 2017). Wheat grain yields can be extremely negatively influenced by abiotic factors like nutrient availability (van der Bom *et al.* 2017) and extreme weather events such as drought, heat, and heavy rainfall (Trnka *et al.* 2014). In addition, biotic constraints like plant pathogens, insects, and weeds can lead to severe yield losses despite the use of fungicides, insecticides, and herbicides (Singh *et al.* 2016; Deutsch *et al.* 2018). Therefore, and especially in times of an increasing demand for sustainable agriculture (European Parliament 2009, Altieri 2018), the development of improved wheat cultivars must address resistance to diseases in addition to primary breeding targets such as grain yield and quality. In Germany, new winter wheat cultivars need to pass a three-year evaluation, including assessment of disease resistance, before achieving registration as a new marketable variety. Among other fungal pathogens, wheat susceptibility to *Blumeria graminis* (the causal agent of powdery mildew, PM), *Zymoseptoria tritici* (septoria tritici blotch, STB), and *Pyrenophora tritici-repentis* (tan spot, TS) is assessed during registration trials (Bundessortenamt 2016).

*B. graminis* is an obligate biotrophic fungus that reproduces only on living cell tissue and infects wheat from the seedling stage to ear emergence. The infection not only adversely impacts quality parameters, due to altered composition of the grain content and depletion of carbohydrate reserves, but also yield components such as thousand kernel weight (Gao *et al.* 2018). Currently, more than 100 PM resistance alleles are described in the literature, distributed over almost all 21 wheat chromosomes (Buerstmayr *et al.* 2016; Li *et al.* 2018). In addition, genetic studies have identified at least 119 quantitative trait loci (QTL) associated with adult plant resistance to PM (Li *et al.* 2014). Despite the large number of known PM resistance genes and QTL, the need to breed new resistant varieties is an ongoing task, as the pathogen possesses a diverse haplotype pool that underpins continual adaption of the fungus to improvements in host genetic resistance (Wicker *et al.* 2013).

STB, caused by the necrotrophic fungus *Z. tritici*, is one of the most damaging fungal diseases in regions with humid climates (Fones and Gurr 2015). Infection via wind-dispersed ascospores, which originate from stubble and wheat volunteers, takes place during early wheat developmental stages (Suffert *et al.* 2011). In a second stage of the fungal life cycle, infection mainly occurs by splash-dispersed pycnidiospores from basal leaf layers (Suffert *et al.* 2011). The long latent period, which typically lasts 3-4 weeks, makes it difficult for farmers to react sufficiently early to STB infection (Orton *et al.* 2011). In addition the intensive use of fungicides for controlling STB increases problems with fungicide resistance. In recent years, target site mutations have been found to evolve in the *Z. tritici* populations to commonly used fungicide groups including azoles, succinate dehydrogenase inhibitors, and strobilurins, providing increasing problems with effective control (Jørgensen *et al.* 2018). Genetic analyses investigating STB resistance have previously identified 21 major genes and at least 89 QTL (Brown *et al.* 2015). Despite this, there is still a lack of a broad range of commercially relevant wheat germplasms which show adequate resistance to *Z. tritici* infection (O’Driscoll *et al.* 2014).

Another economically significant residue-born wheat disease worldwide is TS, caused by the necrotrophic fungus *P. tritici-repentis*. Reduced tillage and retaining stubble, which are partly practices to stabilize the soil in sustainable agricultural approaches, increase the risk of infection due to the presence of *P. tritici-repentis* spores in stubble from previous season crops (Bockus and Shroyer 1998). The main symptoms are yellowish brown leaf spots surrounded by chlorotic and necrotic areas (Lamari and Bernier 1989). Disease development results in coalescence of spots, and consequently in a reduced assimilation rate and yield losses (Faris *et al.* 2013). The symptoms are mainly associated with the three *P. tritici-repentis* necrotrophic fungal protein effectors ToxA, ToxB, and ToxC (Strelkov and Lamari 2003), with further effectors also thought to be involved (Faris *et al.* 2013). Based on the ability of *P. tritici-repentis* to produce these three virulence factors, isolates are classified into eight different races (Strelkov and Lamari 2003). In wheat, allelic variation at the three effector sensitivity loci *Tsn1*, *Tsc1* and *Tsc2*, and the four tan spot resistance loci *Tsr2*, *Tsr3*, *Tsr4*, and *Tsr5* are known to confer qualitative resistance (Faris *et al.* 2013). Additionally, race-nonspecific QTL have been identified for TS resistance (Kollers *et al.* 2014; Juliana *et al.* 2018).

Because new wheat varieties need to be resistant to multiple fungal pathogens, it is of great interest whether simultaneously breeding for resistance to PM, STB, and TS is feasible without taking into account possible genetic interactions between resistance loci for these pathogens. So far, genetic studies in wheat have addressed the relationship of genetic resistance to multiple diseases mainly within bi-parental mapping populations and genome-wide association panels (Lillemo *et al.* 2008; Gurung *et al.* 2012, 2014; Li *et al.* 2014; Jighly *et al.* 2016; Juliana *et al.* 2018). Some describe genetic regions that are associated with multiple disease resistances such as to leaf rust, stripe rust, and PM (Li *et al.* 2014) or leaf rust, stripe rust, and TS (Juliana *et al.* 2018). However, the absence of genetic relations (Gurung *et al.* 2014) and the existence of antagonistic relations between different disease resistances are also propagated (Brown and Rant 2013; Jighly *et al.* 2016).

Simultaneous genetic analysis of resistance to the important wheat fungal diseases PM, STB, and TS at high genetic resolution, which can be achieved using multiparental populations, is of interest for resistance breeding. To address this, we carried out QTL mapping for disease resistance to PM, STB, and TS using a winter wheat multiparent advanced generation intercross (MAGIC) population. The population was constructed using eight founders, which differ in susceptibility to the three target diseases. First, using phenotypic data from MAGIC field trials at six sites over two seasons, we undertook QTL analysis for resistance to PM, STB, TS, and the agro-morphological traits plant height (PH), ear emergence time (EET), and leaf angle distribution (LAD), using four different genetic mapping approaches. Second, we analyzed coinciding QTL based on overlap of support intervals, and explored their relationships. Third, we identified and discuss potential genes underlying the traits of interest, which can serve as a starting point for further studies.

## Material and Methods

### Plant material and genetic map

An eight-founder MAGIC population of winter wheat (termed the ‘BMWpop’) comprising 394 F_6:8_ lines was used (Stadlmeier *et al.* 2018). The eight founders ‘Event,’ ‘BAYP4535,’ ‘Ambition,’ ‘Firl3565,’ ‘Format,’ ‘Potenzial,’ ‘Bussard,’ and ‘Julius’ differed in various agro-morphological and disease traits. A genetic linkage map including 5435 single nucleotide polymorphism (SNP) markers and a functional marker for the powdery mildew resistance gene *Pm3a* was used for QTL analyses (Stadlmeier *et al.* 2018).

### Field trials and trait evaluations

Field trials for assessing reactions to PM, STB, and TS were conducted in Germany and Denmark during 2016 and 2017 (Table 1). The field trial design in each year–location combination was an incomplete block design with two replicates each. The trial at Roggenstein consisted of plots 1.5 m × 3 m in size with ∼1300 plants per plot. All other locations used double rows containing ∼15 plants per row. Each trait was assessed plotwise as the mean of all plants per plot. Thus, trait evaluation is based on 1300 and 30 plants at Roggenstein and the remaining locations, respectively. Founders, control varieties, and checks differing in susceptibility were included with at least two replicates each in all field trials except for Roggenstein. At Roggenstein only founders were included. Fertilization and pest management, except fungicide treatment, followed the standard agronomic procedures at each location.

**Table 1:** Overview of field trial environments and traits scored

Year	Location	GPS*^a^*	Abbreviation	Inoculation	Traits scored*^b^*
**2016**	Freising (DEU)	48°24’45.3”N 11°43’21.1”E	16FS1	*Z. tritici* conidia	PH, EET, LAD, STB
**2016**	Freising (DEU)	48°24’01.8”N 11°42’46.4”E	16FS2	None	PH, EET, LAD, TS
**2016**	Roggenstein (DEU)	48°10’51.9”N 11°19’07.3”E	16RG	None	PM
**2017**	Freising (DEU)	48°24’43.9”N 11°43’20.1”E	17FS1	*Z. tritici* conidia	PH, EET, LAD, PM, STB
**2017**	Freising (DEU)	48°24’38.6”N 11°43’27.9”E	17FS2	*P. tritici-repentis* infected straw	PH, EET, TS
**2017**	Slagelse (DNK)	55°25’06.9”N 11°22’54.7”E	17SL	*P. tritici-repentis* infected straw	PH, EET, LAD, TS

a Global Positioning System (GPS) coordinates.

b Traits are plant height (PH), ear emergence time (EET), leaf angle distribution (LAD), septoria tritici blotch (STB), tan spot (TS), and powdery mildew (PM).

#### Powdery mildew:

Field trials were conducted in Germany during 2016 and 2017 (Table 1). In both years, powdery mildew infection occurred naturally without the use of susceptible varieties as spreaders. No fungicides were used before scoring time point for controlling other fungal diseases. Whole-plot disease severity was scored from 1 (no disease) to 9 (severe disease) according to the guidelines of the German Seed Board (Bundessortenamt 2000, Mohler *et al.* 2013, Table S1) when the ligule of the flag leaf was just visible (Feekes 9).

#### Septoria tritici blotch:

Ten *M. graminicola* single-spore isolates, collected by the company EpiLogic GmbH (Freising, Germany) across Germany in 2015, and two historical isolates BAZ 6/1/04 and BAZ 8/8/04 (Risser *et al.* 2011), kindly provided by the Julius-Kühn-Institute (Quedlinburg, Germany), were screened for strong isolate growth and sporulation on media and virulence to all eight founders of BMWpop at the seedling stage. Single spore isolates were cultivated on yeast-glucose-malt agar (4 g yeast, 4 g malt, 4 g glucose, and 15 g agar per liter of distilled water) for five to seven days under white and ultraviolet light for 16 h at 20° and 8 h in darkness at 12° in an MLR-351 incubator (Sanyo Electric Co., Osaka, Japan). These plates were used as starter cultures for mass propagation in liquid yeast-glucose-malt medium (4 g yeast, 4 g malt, and 4 g glucose per liter of distilled water) as described in Risser *et al.* (2011). Fresh conidia of the isolates were concentrated using a separating funnel. The conidia concentration was determined using a Fuchs-Rosenthal hemocytometer (depth 0.2 mm; Laboroptik GmbH, Friedrichsdorf, Germany). The inoculum concentration was adjusted to 1x10^6^ conidia/ml consisting of equal shares of the four isolates St-SN-002, St-SN-004, BAZ 6/1/04, and BAZ 8/8/04. The inoculum was supplemented with 0.05% (v/v) Tween80. The BMWpop for analysis of STB resistance was cultivated in Germany in 2016 and 2017 (Table 1). In 2016, two weeks before inoculation the fenpropimorph fungicide Corbel was applied preventively with a reduced spray rate of 0.75 l per hectare. In 2017, seven days before inoculation a reduced application rate of 0.5 l per hectare was applied. The fungicidal activity of Corbel is against yellow and brown rust and powdery mildew (Anonymous 2018). The trials were artificially inoculated when the sheath of the flag leaf was completely grown out (Feekes 10-10.1). Inoculation was carried out two times at one-week intervals with 660 l inoculum per hectare during cloudy afternoons. An hour after the first inoculation, mist irrigation was applied with a one-hour break for each two hours of irrigation from 8:00 am to 6:00 pm for the duration of eleven days. Six weeks after inoculation when the trait of interest showed a maximum of differentiation within the population, disease development on the flag leaf was assessed plotwise. Scoring was based on visually assessing the percentage leaf area covered by lesions exhibiting pycnidia using a rating scale from 0% (no symptoms) to 100% (severe disease).

#### Tan spot:

In 2016, a field trial was carried out at a location in Germany where wheat after wheat was sown for over ten years with reduced tillage. Two additional field experiments were conducted in Denmark and Germany in the 2017 cropping season (Table 1). The experiments in the second year were both inoculated with 50 g/m^2^ naturally *P. tritici-repentis* infected straw. The spreading of this straw litter took place in Denmark in December 2016 and in Germany in March 2017 when two to five leaves were unfolded (Feekes 1.2-1.5). In 2016 and 2017 in Germany, the fenpropimorph fungicide Corbel was applied preventively with a reduced spray rate of 0.75 and 0.5 l per hectare, respectively, before Feekes growth stage 8. In Denmark, the trial was treated with a reduced spray rate of 0.5 l pyraclostrobin fungicide Comet 200 per hectare at Feekes growth stage 7.1-7.4 to prevent the trial from rust infection. It is known that the level of resistance to the strobilurins fungicides within the *P. tritici-repentis* population is very high in the Danish test population and therefore, the control of tan spot using Comet 200 has to be considered as insignificant (Sierotzki *et al.* 2007). Two weeks after the first symptoms appeared, the infection of the flag leaf was visually assessed based on the percentage leaf area exhibiting brown to black spots surrounded by necrotic and chlorotic areas using a rating scale from 0% (no infestation) to 100% (severe disease).

#### Agro-morphological traits:

The three agro-morphological traits, plant height, ear emergence time, and leaf angle distribution, were assessed in almost all trials (Table 1). PH was measured in cm from ground level to the top of the ears (excluding scurs) from Feekes developmental stage 11.3 to the end of cropping season. EET was recorded when half of the ear emerged above the flag leaf ligule (Feekes 10.3) for half of all plants per plot and converted into number of days after 1^st^ May. The LAD score combined the leaf angle between the stem and the proximal third of the leaf and the overhanging of the distal part of the leaf. The rating was carried out in odd numbers from 1 (erectophile, <45°; no overhanging) to 9 (planophile, >45°; overhanging) at anthesis (Feekes 10.51-10.53).

### Phenotypic data analysis

Outlying observations were identified within the raw phenotypic datasets according to Grubbs (1950) and removed only under obvious incorrect scoring or known problems with the specific plot. Each year–location combination was treated as an individual test environment (TE). All phenotypic data analysis were performed using R/lme4 (Bates *et al.* 2014; R Development Core Team 2017). The residuals of all traits in individual and across TEs were analyzed with residual diagnostic plots. Phenotypic data analysis and repeatability in individual TEs was based on a reduced model (equation 1) without consideration of the TE and the genotype * TE interaction effect. Phenotypic data across the TEs were adjusted based on the following model:

$$y_{ijkm}=\mu +g_{i}+l_{j}+gl_{ij}+r_{kj}+b_{mkj}+e_{ijkm}.$$ (1)

where $y_{ijkm}$ is the trait observation, μ is the overall mean, $g_{i}$ is the fixed effect of genotype i, $l_{j}$ is the random effect of TE j, $gl_{ij}$ is the random interaction effect of genotype i with TE j, $r_{kj}$ is the random effect of replication k nested within TE j, $b_{mkj}$ is the random effect of incomplete block m nested within replication k nested within TE j and $e_{ijkm}$ is the random residual error. To obtain variance components, the genotype was fitted as random. The repeatability was the ratio of the genotypic variance and the sum of the genotypic and residual error variance. The heritability was estimated on an entry mean basis according to Hallauer and Miranda (1981). The Pearson’s phenotypic correlation between traits was calculated based on the adjusted means across the TEs. Genotypic correlation coefficients were calculated in PLABSTAT Version 3Awin (Utz 2011) using adjusted means from common individual TEs and fitting genotype and TE as random.

### QTL analysis

**Figure 1:**
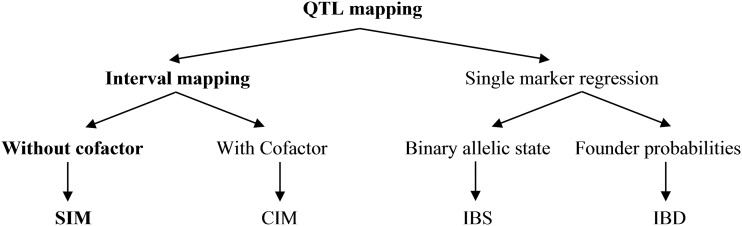
Overview of the QTL detection methods used.

The adjusted means estimated in the individual TEs and in the combined analysis across TEs represented the phenotypic input data for QTL detection. The simple interval mapping (SIM) approach implemented in the package mpMap V2.0.2 (Huang and George 2011) was applied as the main QTL detection method (Figure 1). A subset of 2804 markers representing unique positions on the BMWpop genetic map (Stadlmeier *et al.* 2018) was used in interval mapping. The QTL mapping was based on founder probabilities computed using the mpMap function ‘mpprob’ implemented in R/mpMap (Huang and George 2011) at a threshold of 0.7. The function ‘mpIM’ was used for interval mapping. QTL were detected at a genome-wide significance threshold of α < 0.001. This threshold was derived from an empirically null distribution with 1,000 simulation runs similar to Churchill and Doerge (1994). All detected QTL were simultaneously fitted in a full model using the function ‘fit.’ From this model fit, only QTL were kept with a p-value < 0.10 and the full model was fitted again to obtain the phenotypic variance explained (R^2^) and the additive founder effects relative to ‘Julius.’ The QTL support interval (SI) was determined as the map distance in cM surrounding a QTL peak at a -log10(p) fall-off of ±1.0. If QTL SIs overlapped, QTL were declared coinciding QTL (cQTL). QTL detected in SIM analysis were named following the recommendations for gene symbolization in wheat (McIntosh *et al.* 2013). To determine the physical position of each QTL in the wheat reference genome assembly, the sequence of the peak marker was used as a query against the IWGSC RefSeq v1.0 genome assembly (International Wheat Genome Sequencing Consortium 2018) using BLASTn analysis (Altschul *et al.* 1990). For composite interval mapping (CIM) (Figure 1), only linkage groups found to be significant in SIM were used. The CIM analysis was based on a forward selection procedure using the Akaike information criteria (Huang and George 2011). The number of selected cofactors was set to the number of detected QTL in SIM.

Another QTL mapping approach, a single marker analysis similar to Sannemann *et al.* (2015), was addressed to support the outcome of simple interval mapping. For this approach, all 5436 available markers were integrated. Generally, two different methods were used (Figure 1). In the identical by state (IBS) method, a regression against the binary allelic state (homozygote allele A, homozygote allele B) of each marker was conducted and the additive effect of the minor allele was estimated. In the identical by descent (IBD) method, a regression was conducted against the founder probabilities of each marker. Founder probabilities were calculated as stated above, and additive founder effects were estimated relative to ‘Julius.’ In both single marker regression methods, a random effect for MAGIC group assignment of each genotype was included initially. When this term was not significant, it was excluded from the final analysis. The derived p-values were corrected for multiple testing according to Benjamini and Hochberg (1995). Marker-trait associations (MTAs) were declared significant at a false discovery rate (FDR) of 0.01. Adjacent significant SNPs on a linkage group were assumed to represent the same QTL whenever the direction of additive effects was the same. Further QTL on the same linkage group were accepted when a MTA was at least 30 cM from another one. The MTA with the most significant FDR value was chosen as the QTL peak position. Finally, a full model including all detected QTL was fitted to estimate the overall R^2^.

A QTL in SIM analysis was declared detected with other approaches when the SIs overlapped.

### Data availability statement

Genotypic data are publically available at http://doi.org/10.14459/2018mp1435172. File Figure S1 contains the distribution of phenotypic data within test environments. File Figure S2 represents the residual diagnostic plots of phenotypic data. File Figure S3 shows the density plots of adjusted means. Supplementary Table S1 shows the rating scale for PM. Supplementary Tables S2 and S3 represent the phenotypic data within and across test environments, respectively. Supplementary Table S4 contains the correlation coefficients. Files Table S5-S8 show detailed mapping results for all traits analyzed. Supplemental material available at Figshare: https://doi.org/10.25387/g3.7862531.

## Results

### Quantitative genetic analysis

The repeatability of trait measurements in the field trials ranged from 0.57 to 0.92 (Table 2). The correlation of the adjusted means (Table S2) between TEs was significant (*P* < 0.001) for each trait (Table 2). The highest values were observed for PH (0.93) and EET (0.84), while for the remaining traits the correlations ranged from 0.28 (LAD) to 0.63 (STB). The severity of infection between the TEs differed significantly at *P* < 0.05 for all three disease traits (Figure S1). The residuals of the adjusted means across TE’s (Table S3) followed approximately a normal distribution (Figure S2). Box-Cox and log transformations were applied but did not improve the normality and homoscedasticity of the data. Therefore, untransformed data was used for all analyses. The population mean was not significantly different from the parental mean for all traits (Table 2). However, transgressive segregation was identified for all traits except for STB (Figure S3). The full range of reaction scores was found for PM and STB compared to TS, for which the range was from 17 to 75% (Figure S3). The genotypic and genotype * TE variance components were significant at *P* < 0.01 for all traits in the combined analysis across TEs (Table 2). The heritability estimates for PM, STB, TS, and LAD were 0.64, 0.79, 0.70, and 0.77, respectively (Table 2); they were high for PH and EET with 0.98 and 0.93, respectively. Phenotypic data for STB was significantly correlated with PM and TS with 0.22 and 0.33, respectively (Table S4). However, the latter two diseases did not correlate phenotypically. PH and EET revealed significant negative phenotypic and genotypic correlation coefficients with STB and TS (Table S4). Although LAD showed no significant phenotypic correlations, genotypic correlations with STB and TS were significant with 0.32 and -0.20, respectively.

**Table 2:** Estimates of the repeatability, the phenotypic correlation between test environments (TEs), the mean (± SE) of the population, the range of the population, the mean (± SE) of the founders, the genetic, the genotype * TE interaction, and the residual variance component (${\hat{\sigma }}_{g}^{2}$, ${\hat{\sigma }}_{\mathrm{gl}}^{2}$, ${\hat{\sigma }}_{e}^{2}$), the heritability (${\hat{h}}^{2}$), and the number of test environments (No.TE) of the traits investigated

Trait*^a^*	No.TE	Repeatability	Cor(TE_x_,TE_y_)**	Mean(pop)	Range(pop)	Mean(found)	${\hat{\sigma }}_{g}^{2}$*	${\hat{\sigma }}_{\mathrm{gl}}^{2}$*	${\hat{\sigma }}_{e}^{2}$	${\hat{h}}^{2}$
**PM**	2	0.57, 0.75	0.56	2.8 ± 0.08	0.6 - 7.7	2.5 ± 0.53	1.6	1.2	1.2	0.64
**STB**	2	0.83, 0.85	0.63	38.8 ± 0.88	8.5 - 91.5	36.8 ± 9.30	253.2	104.4	68.0	0.79
**TS**	3	0.39 - 0.67	0.34 - 0.61	38.6 ± 0.52	16.5 - 74.8	36.3 ± 3.09	74.4	42.4	102.6	0.70
**PH**	5	0.85 - 0.92	0.87 - 0.93	94.9 ± 0.42	72.3 - 113.4	90.3 ± 2.45	82.1	3.9	9.0	0.98
**EET**	5	0.67 - 0.85	0.60 - 0.84	34.8 ± 0.09	30.2 - 41.6	35.4 ± 0.49	3.6	0.6	1.1	0.94
**LAD**	4	0.44 - 0.59	0.28 - 0.49	5.3 ± 0.07	1.8 - 8.5	5.2 ± 0.30	1.3	0.7	1.8	0.77

a Powdery mildew (PM, score 1-9), septoria tritici blotch (STB, %), tan spot (TS, %), plant height (PH, cm), ear emergence time (EET, DaM), and leaf angle distribution (LAD, score 1-9).

* Significant at *P* < 0.01.

** Significant at *P* < 0.001.

### QTL mapping

**Figure 2:**
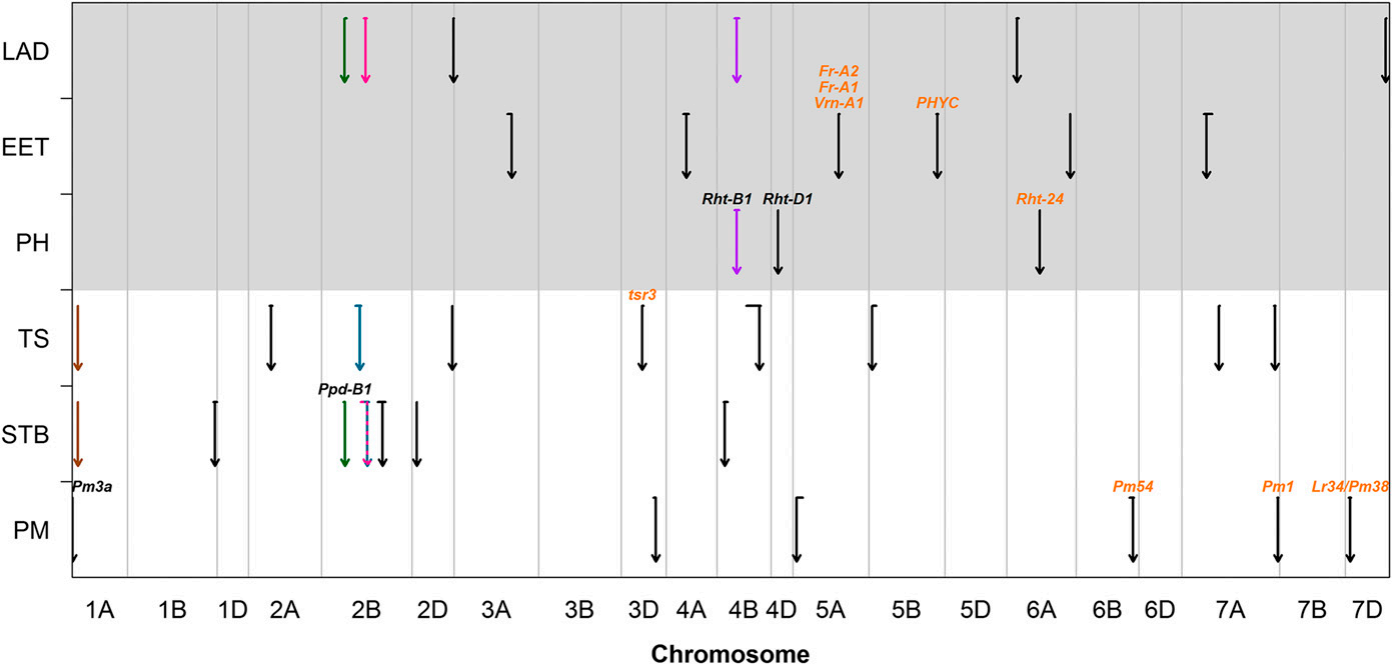
QTL detected in SIM analysis across test environments. The results are shown for the traits powdery mildew (PM), septoria tritici blotch (STB), tan spot (TS), plant height (PH), ear emergence time (EET) and leaf angle distribution (LAD). The arrows indicate the exact localization on the chromosome and the horizontal line above each arrow represents the support interval (SI) of the respective QTL. Overlapping QTL are labeled using the same color. Gene assignments based on functional markers are indicated in black and gene assignments based on physical positions are indicated in orange.

SIM analysis of the disease and agro-morphological traits identified 22 and 15 QTL across TEs, respectively (Figure 2 and Table 3). The number of detected QTL for individual traits was between three (PH) and nine (TS). For each trait, the QTL collectively explained about 30% of the total phenotypic variance except for TS and PH with a total R^2^ value of 40.5% and 53.0%, respectively (Table 3). In general, all mapping approaches explained comparable proportions of the phenotypic variance, although the IBS method always showed slightly lower values. An overview of the QTL detection with SIM is shown in Table 3 and detailed information including allelic effects and the detection within single TEs is given in the Table S5. The results of CIM, IBD, and IBS analysis are presented in Table S6, S7, and S8, respectively. The below-mentioned additive allelic effects are stated relative to the founder ‘Julius’.

**Table 3:** Summary of QTL detected in SIM analysis across test environments. Chromosome (Chr.), position (Pos.), support interval (SI), -log(p) value, proportion of phenotypic variance explained (R^2^), and the number of TEs (No.TE) in which a QTL was detected are given. Additionally, it is indicated whether QTL were also detected by composite interval mapping (CIM), identical by descent (IBD), and identical by state (IBS) approaches

Trait*^a^*	QTL	Chr.	Pos. (cM)	Pos (Mbp)	SI (cM)	-log(p)	R^2 b^	No.TE	Method
**PM [1-9]**							**31.5**		
	*QPm.lfl-1A*	1A	3	**–**	2-3	6.4	9.4	1	CIM, IBD, IBS
	*QPm.lfl-3D*	3D	137	570	128-137	1.9	4.8	—	—
	*QPm.lfl-5A*	5A	16	19	14-38	1.2	4.9	—	—
	*QPm.lfl-6B*	6B	227	710	211-230	4.4	6.7	2	CIM, IBD, IBS
	*QPm.lfl-7A*	7A	382	730	379-384	5.2	12.8	2	CIM, IBD, IBS
	*QPm.lfl-7D*	7D	19	**–**	12-22	1.0	5.3	—	IBD
**STB [%]**							**27.1**		
	*QStb.lfl-1A*	1A	23	9	23-23	1.2	6.1	—	IBD
	*QStb.lfl-1B*	1B	347	690	341-356	2.2	5.9	1	CIM
	*QStb.lfl-2B.1*	2B	94	57	86-94	1.1	5.3	1	IBD
	*QStb.lfl-2B.2*	2B	181	440	155-188	1.2	4.7	1	—
	*QStb.lfl-2B.3*	2B	243	700	222-254	3.5	6.3	2	CIM, IBD, IBS
	*QStb.lfl-2D*	2D	20	10	20-20	5.4	8.5	2	CIM, IBD, IBS
	*QStb.lfl-4B*	4B	31	24	27-43	1.5	4.3	—	—
**TS [%]**							**40.5**		
	*QTs.lfl-1A*	1A	23	9	23-23	5.1	6.8	—	CIM, IBD
	*QTs.lfl-2A*	2A	90	71	83-94	4.7	6.6	1	CIM, IBD, IBS
	*QTs.lfl-2B*	2B	151	130	135-157	3.9	4.6	—	IBD
	*QTs.lfl-2D*	2D	160	640	160-160	4.5	6.7	2	IBD, IBS
	*QTs.lfl-3D*	3D	82	210	81-88	4.4	7.1	2	CIM, IBD
	*QTs.lfl-4B*	4B	170	600	117-175	1.6	5.2	—	IBD
	*QTs.lfl-5B*	5B	28	41	28-31	2.5	5.0	—	IBD, IBS
	*QTs.lfl-7A.1*	7A	148	130	148-151	3.5	10.6	2	CIM, IBD, IBS
	*QTs.lfl-7A.2*	7A	371	710	366-373	3.5	4.5	—	—
**PH [cm]**							**53.0**		
	*QHt.lfl-4B*	4B	80	63	68-90	∞	9.2	5	CIM, IBD, IBS
	*QHt.lfl-4D*	4D	27	**–**	27-27	∞	33.6	5	CIM, IBD, IBS
	*QHt.lfl-6A*	6A	130	450	130-130	10.8	12.6	2	IBD, IBS
**EET [DaM]**				0			**25.9**		
	*QEet.lfl-3A*	3A	230	650	213-230	4.2	8.8	5	CIM, IBD, IBS
	*QEet.lfl-4A*	4A	80	560	67-89	1.9	5.2	1	—
	*QEet.lfl-5A*	5A	182	550	180-185	3.5	7.6	4	CIM, IBD, IBS
	*QEet.lfl-5B*	5B	273	690	268-276	1.5	5.7	1	—
	*QEet.lfl-6A*	6A	251	600	250-252	2.9	5.0	1	IBS
	*QEet.lfl-7A*	7A	99	85	87-122	2.0	5.5	3	IBD
**LAD [1-9]**							**32.3**		
	*QLad.lfl-2B.1*	2B	92	53	92-100	1.4	5.8	—	—
	*QLad.lfl-2B.2*	2B	174	190	167-180	9.0	14.4	2	CIM, IBD, IBS
	*QLad.lfl-2D*	2D	166	640	164-167	3.7	5.6	1	CIM, IBD, IBS
	*QLad.lfl-4B*	4B	80	90	70-89	2.5	6.4	1	IBD, IBS
	*QLad.lfl-6A*	6A	40	19	36-46	4.7	6.6	1	CIM, IBD
	*QLad.lfl-7D*	7D	160	600	160-163	2.3	4.6	1	—

a Powdery mildew (PM), septoria tritici blotch (STB), tan spot (TS), plant height (PH), ear emergence time (EET), and leaf angle distribution (LAD).

b For each trait, the explained phenotypic variance of the model fitting all detected QTL simultaneously is given in bold values above the individual R^2^ values for each QTL.

- No marker sequence for BLASTn query was available.

— QTL in no test environment or with no other method detected.

∞ P-value is zero.

#### Powdery mildew:

The QTL analysis of PM across TEs identified six QTL, which were located on chromosomes 1A, 3D, 5A, 6B, 7A, and 7D (Figure 2 and Table 3). The most significant QTL *QPm.lfl-1A* accounted for 9.4% of the phenotypic variance and coincided with the functional marker for the *Pm3a* gene. The resistance allele was contributed by the parent ‘BAYP4535’ which is the only founder carrying this allele (Stadlmeier *et al.* 2018). Two other highly significant QTL on chromosomes 7A and 6B explained 12.8% and 6.7% of the phenotypic variance, respectively. On chromosome 7A, the founders ‘Event’ and ‘Ambition’ and on chromosome 6B, the founder ‘Ambition’ decreased disease severity. Both QTL were identified by all mapping approaches and in all TEs (Table S5-S8).

#### Septoria tritici blotch:

Seven QTL controlling resistance to STB were mapped to chromosomes 1A, 1B, 2B, 2D, and 4B in the analysis across TEs (Figure 2 and Table 3). The two most significant QTL *QStb.lfl-2B.3* and *QStb.lfl-2D*, which were identified in all TEs and with all detection methods (Table S5-S8), explained 8.5% and 6.3% of the phenotypic variance, respectively (Table 3). At *QStb.lfl-2B.3*, the alleles of the founders ‘Ambition’ and ‘Firl3565’ decreased disease severity by 10.1% and 14.1%, respectively (Table S5). The ‘Format’ allele at *QStb.lfl-2D* increased disease severity by 10.1% (Table S5). The three QTL *QStb.lfl-1B*, *QStb.lfl-2B.1*, and *QStb.lfl-2B.2* were detected additionally to the combined analysis across environments in one of two TEs (Table S5). The peak marker of *QStb.lfl-2B.1* corresponded to a functional marker diagnostic for the late-heading allele at the photoperiod response gene, *Photoperiod-B1* (*Ppd-B1*).

#### Tan spot:

For TS, nine QTL across environments were detected on chromosomes 1A, 2A, 2B, 2D, 3D, 4B, 5B, and 7A (Figure 2 and Table 3). The most significant QTL *QTs.lfl-1A* explained 6.8% of the phenotypic variation for this trait. The ‘Bussard’ allele increased TS infestation by 14.1% at this locus (Table S5). The QTL *QTs.lfl-7A.1*, *QTs.lfl-3D*, and *QTs.lfl-2D* also explained high proportions of the phenotypic variance of 10.6%, 7.1%, and 6.7%, respectively. These QTL were identified using several mapping approaches, and in two of three TEs each (Table 3 and Table S5-S8). The QTL *QTs.lfl-2A* on chromosome 2A explained 6.6% of the phenotypic variance and was mapped across TEs by all analysis methods and in one of three TEs (Table S5-S8).

#### Agro-morphological traits:

Three PH QTL were identified on chromosomes 4B, 4D, and 6A (Figure 2 and Table 3). The two most significant QTL, *QHt.lfl-4D* and *QHt.lfl-4B*, explained 33.6% and 9.2% of the phenotypic variance, respectively (Table 3). The SIs of *QHt.lfl-4D* and *QHt.lfl-4B* coincided with the dwarfing genes *Reduced height-D1* (*Rht-D1*) and *Rht-B1*, respectively. The effects of the founders ‘BAYP4535,’ ‘Firl3565,’ ‘Format,’ and ‘Bussard,’ all carriers of the wild-type *Rht-D1a* allele, increased PH by up to 29 cm. The mildly dwarfing *Rht-B1b* allele possessed by ‘BAYP4535’ decreased PH by 9.9 cm (Table S5). The QTL *QHt.lfl-6A* was also highly significant and explained 12.6% of the phenotypic variation for this trait. Alleles from ‘Ambition’ and ‘Potenzial’ decreased PH by more than 9 cm at this QTL (Table S5).

The QTL analysis for EET detected six QTL on chromosomes 3A, 4A, 5A, 5B, 6A, and 7A (Figure 2 and Table 3). The two most significant QTL, *QEet.lfl-3A* and *QEet.lfl-5A*, explained 8.8% and 7.6% of the phenotypic variance, respectively. They were mapped with all approaches and in almost all TEs (Table S5-S8). *QEet.lfl-3A* alleles from ‘Format,’ ‘Firl3565,’ and ‘Ambition’ delayed EET by up to 1.6 days, while at *QEet.lfl-5A* the ‘Bussard,’ ‘Potenzial,’ and ‘BAYP4535’ alleles delayed EET by up to 1.5 days (Table S5). The QTL *QEet.lfl-7A* was observed in three of five TEs, explaining 5.5% of the phenotypic variance (Table 3 and Table S5).

Six QTL controlling LAD were identified on chromosomes 2B, 2D, 4B, 6A, and 7D (Figure 2 and Table 3). The most significant QTL *QLad.lfl-2B.2* explained 14.4% of the phenotypic variation and was identified by all methods (Table 3 and Table S5-S8). The ‘Event’ allele at this locus was associated with a more erectophile LAD (Table S5). The QTL *QLad.lfl-2D*, which explained 5.6% of the phenotypic variance, was also observed by all mapping approaches (Table 3 and Table S5-S8). The allele, which was associated with a more erectophile LAD, originated from ‘Bussard’ and showed an effect of 2.3 grades (Table S5).

### Coinciding QTL

In total, five cQTL were identified on chromosomes 1A, 2B, and 4B (Figure 2). Two QTL overlaps each were detected for STB – TS and STB – LAD. One QTL overlap was observed on chromosome 4B for PH and LAD. The perfect overlap of *QStb.lfl-1A* and *QTs.lfl-1A* on chromosome 1A was pinpointed to 23 cM of the genetic map (Table 3). The ‘Bussard’ allele increased both STB and TS severity by 11.6% and 14.1%, respectively. The allelic effects of the remaining parents were not clearly opposite, except for the founder ‘Event’ (Table S5). The second cQTL between STB and TS was located on chromosome 2B. *QStb.lfl-2B.2* and *QTs.lfl-2B* showed just a small overlap of 2 cM and the respective peak markers were located 30 cM away from each other (Table 3). The alleles of ‘BAYP4535’ and ‘Ambition’ decreased both STB and TS severity by up to 6.1% and 10.8%, respectively. However, the alleles of the founders ‘Event’ and ‘Potenzial’ showed strong opposite effects with a difference of over 20% for the two diseases (Table S5). On chromosome 2B, an overlap between the already mentioned QTL *QStb-lfl.2B.2* for STB and the QTL *QLad.lfl-2B.2* for LAD was present. The SI of the LAD QTL with a size of 13 cM was completely embedded in the other one (Table 3). Especially the allelic effects of ‘Event’ revealed that a decrease in STB infestation came along with a more erectophile LAD but also the effects of the parents ‘Ambition,’ ‘Potenzial,’ and ‘Bussard,’ showed this behavior (Table S5). A second cQTL for STB and LAD was identified on the short arm of chromosome 2B, and coincides with the location of the major photoperiod response locus *Ppd-B1*. Although there was just a small overlap of 2 cM between *QStb.lfl-2B.1* and *QLad.lfl-2B.1*, the peak markers were located just 2 cM away from each other (Table 3). Despite this fact, the allelic effects showed no clear relational pattern of STB and LAD (Table S5). A further cQTL was located on chromosome 4B for PH and LAD. The SI of *QLad.lfl-4B* completely overlapped with the SI of *QHt.lfl-4B*. The allele of the founder ‘BAYP4535’, which is carrier of the *Rht-B1b* dwarfing allele, reduced height and was associated with a more planophile LAD. The alleles of the other parents showed no distinct relations (Table S5).

## Discussion

### Field trials

In total, six field trials for the three diseases PM, STB, and TS were conducted. Except for the trial 17FS1, in each trial, a single disease was assessed and additional infections with other leaf diseases were not observed. In 17FS1 PM and STB were scored in the same field experiment but at different times. After scoring PM and several days before inoculation with STB, a fungicide against PM was applied. Although PM was scored in the STB trial in 2017, no genotype was so heavily infected that individual plants died before STB infestation. However, it appears unlikely that PM infestation had an influence on STB disease development, as the phenotypic correlation between PM scored in 16RG and STB scored in 16FS1 and 17FS1 was similar with 0.14 and 0.12, respectively. In addition, the correlation between PM scored in 17FS1 and STB scored in 16FS1 and 17FS1 was similar at 0.23 and 0.19, respectively.

### Comparison of applied QTL mapping approaches

In parallel with the development of multiparental population designs, appropriate statistical methods have also been developed to analyze such populations accurately. In addition to advanced mixed linear model approaches for genome-wide association mapping (Bandillo *et al.* 2013; Sannemann *et al.* 2015), various software packages for interval mapping such as R/HAPPY (Mott *et al.* 2000), R/mpMap (Huang and George 2011), R/mpwgaim (Verbyla *et al.* 2014), and R/qtl2 (Broman *et al.* 2019) are now available. Zhang *et al.* (2017) introduced a two-stage QTL mapping strategy for four-way crosses to control the background genetic variation.

We mapped QTL with the single marker regression methods IBD and IBS and with the interval mapping approaches SIM and CIM implemented in R/mpMap (Figure 1). Although the statistical power of CIM is stronger than of SIM, we chose to use SIM as the main QTL detection method as cofactor selection in CIM remains challenging (Li *et al.* 2007; Wei and Xu 2016). We tried to counteract this issue by including only significant linkage groups of SIM in CIM and by setting the number of cofactors equal to the number of detected QTL in SIM. Our results (Table S6) suggest that this methodology is robust and can deal with this issue although it may result in missing of QTL only detectable in CIM.

The total number of QTL mapped with the different approaches strongly varied with the trait. An increased number of QTL for TS (IBS: 17, IBD: 23) and a reduced number of QTL for PM (IBS: 3, IBD: 5) and STB (IBS: 3, IBD: 5) were detected in single marker analysis compared to interval mapping. The IBS method identified 49% and 52% and the IBD method 70% and 68% of the SIM and CIM QTL, respectively. It is known that multiple testing increases the probability of detecting false positives when the statistical significance is not adjusted properly. For IBS and IBD method, we chose a FDR of 0.01 for all traits of interest. Therefore, our results indicate that a trait-specific significance threshold that accounts for the genetic architecture of the respective trait would have been more appropriate for IBS and IBD mapping.

The SIs of IBS and both interval mapping approaches were comparable (as measured in cM), whereas the SIs of IBD were generally longer (Table S7). For the single marker analysis, greater SIs were similarly detected by Sannemann *et al.* (2015) when using haplotype probabilities instead of the allelic state. They suggested that this is due to the haplotype blocks that expand over a genetic distance of several cM. These results indicate that IBD SIs might remain larger, until small haplotype blocks are generated by the mating design and the statistical methods used.

In general, the estimated effects based on founder probabilities were greater than the estimates based on the allelic state. This observation has previously been noted in a barley MAGIC population using a mixed linear model approach with an incorporated multi-locus procedure (Sannemann *et al.* 2015). In addition, we found out that the additive effects in linkage analyses are generally stronger than in IBD. As an example, at *QHt.lfl-4D*, the difference of the additive effects of PH between the two most contrasting parents ‘Event’ and ‘Firl3565’ was 31.7 cm, 32.3 cm, 26.5 cm, and 12.5 cm for SIM, CIM, IBD, and IBS, respectively (Table S5-S8). This raises the question whether the effects estimated using IBD are underestimated, or whether the effects of the linkage analysis are overestimated. Linkage analysis in bi-parental populations provides an unbiased estimate of the QTL effect compared to single-point analysis due to simultaneously analyzed linked markers (Lander and Botstein 1986). Therefore, it is more likely that the additive effects of interval mapping in MAGIC designs are unbiased, and that the haplotype-based founder probabilities are insufficient for estimating unbiased QTL effects in single-point analyses.

Despite the weaknesses of each method, strong and environmentally stable QTL were in most instances mapped with all approaches used here. This indicates that the combined use of these different methods may act as a tool to find robust QTL.

### Strength and weakness of multiparental populations in QTL mapping

MAGIC designs are characterized by a high number of recombination events due to several rounds of inter-crossing (Kover *et al.* 2009; Cockram and Mackay 2018; Stadlmeier *et al.* 2018). Therefore, the probability of recombination between linked genetic loci is increased (Holland 2015). Smaller linkage blocks lead to higher accuracy and smaller SIs in linkage mapping (Li *et al.* 2005). In this study, 38% of the detected QTL in SIM and 60% of the QTL mapped in CIM showed SIs ≤ 5 cM. QTL results of other advanced intermated populations support our findings (Balint-Kurti *et al.* 2007; Huang *et al.* 2010). However, some QTL studies in bi-parental mapping populations also reported small SIs (0-20 cM) (Windju *et al.* 2017; Ren *et al.* 2017; Jia *et al.* 2018). Additionally, higher recombination rates have been suggested to break the linkage of two small-effect QTL, which subsequently may complicate their detection individually (Huang *et al.* 2010). Despite this potential drawback of MAGIC populations, we were able to map QTL with < 5% of the explained phenotypic variance and minor additive effects such as *QPm.lfl-5A*, *QTs.lfl-2A*, and *QTs.lfl-2B* in addition to large QTL with high R^2^ values and high additive effects (Table 3 and Table S5). A QTL study analyzing seed size and seed number using *Arabidopsis thaliana* MAGIC lines also did not observe reduced ability to detect small effect QTL (Gnan *et al.* 2014). Another concern stated about the statistical power of linkage mapping in MAGIC is related to a possible decrease in the number of QTL detectable due to the complex genetic background (Keurentjes *et al.* 2011). Some studies using multiparental populations detected fewer QTL compared to linkage analysis in bi-parental populations for the same trait (Kover *et al.* 2009; Huang *et al.* 2011). However, other studies have found no differences in the number of QTL detected (Gnan *et al.* 2014). Other studies have reported the detection of new QTL, likely due to the increased genetic variation introduced by several founders (Sannemann *et al.* 2015; Pascual *et al.* 2015). For the three diseases PM, STB, and TS, we mapped six, seven and nine QTL using SIM, respectively, and a slightly lower number using CIM. The QTL number for each trait is comparable to bi-parental linkage analyses that reported in the most cases 2-6 QTL for PM (Chantret *et al.* 2001; Börner *et al.* 2002; Bougot *et al.* 2006; Qu *et al.* 2018), 3-10 QTL for STB (Eriksen *et al.* 2003; Risser *et al.* 2011; Tabib Ghaffary *et al.* 2012; Naz *et al.* 2015; Adhikari *et al.* 2015) and 3-7 QTL for TS (Friesen and Faris 2004; Faris and Friesen 2005; Chu *et al.* 2008, 2010; Zwart *et al.* 2010). One study for a segregating wheat x spelt population described eleven QTL for PM but this was because of using a population constructed from a cross between two subspecies (Keller *et al.* 1999). In addition, the number of QTL for PH (3 in SIM and CIM) and EET (6 in SIM and 5 in CIM) was similar to other studies (Börner *et al.* 2002; Klahr *et al.* 2007; Zhang *et al.* 2008; Bai *et al.* 2013). For LAD, no comparison was possible, as this trait has not been widely studied in wheat. Based on these results, we did not find a reduction in power for detecting QTL in multiparental populations. However, especially for PM and STB, we observed a partially strongly reduced proportion of the total explained phenotypic variance of up to 50% compared to the total R^2^ value in bi-parental populations. This could not be explained by the number of detected QTL as it was similar between the studies (Chantret *et al.* 2001; Eriksen *et al.* 2003; Friesen and Faris 2004; Li *et al.* 2005; Bougot *et al.* 2006; Risser *et al.* 2011). R^2^ values depend on both the phenotypic variance, and on the genetic variance. The genetic variance is a function of the QTL effect and the allele frequency of the QTL (Falconer and Mackay 1996). Simulation studies demonstrated that a low minor allele frequency decreases the estimated proportion of the explained phenotypic variance of a QTL independently from the true R^2^ value, given that the QTL effect is constant (Uemoto *et al.* 2015). Unlike the case in bi-parental populations, there is a higher probability for markers of lower minor allele frequencies in MAGIC designs. In the extreme case, only one founder contributes a specific allele to the population, which leads to a minor allele frequency of 1/8 in an eight-founder MAGIC design. This may be responsible for the decreased explained phenotypic variances observed in multiparent crosses. Therefore, it seems obvious that mapping rare QTL variants combined with small effects will be more challenging in MAGIC populations (Kover *et al.* 2009).

### Identification of candidate genes and multitrait loci

To find out whether the QTL detected in this study possibly represent known genes and genetic loci, the physical positions of the QTL peak markers in BMWpop and the markers linked to mapped genes were compared (Figure 2).

*QPm.lfl-7D* was mapped to the same genetic region as the multi-resistance gene *Lr34/Yr18/Sr57/Pm38*. However, the QTL peak marker was located 34 Mbp distal to the race non-specific gene *Lr34* (Krattinger *et al.* 2009). However, it is likely that *QPm.lfl-7D* corresponds to the gene *Lr34*: first, based on parental information (Serfling *et al.* 2011), we know that the parent ‘Potenzial’ possesses this gene, and second, *QPm.lfl-7D* was not mapped in seedling stage (Stadlmeier *et al.* 2018). This is in agreement with the fact that *Lr34* is classified as an adult plant resistance gene. *QPm.lfl-7A* was mapped on the long arm of chromosome 7A. In this region, a number of PM resistance loci have been previously mapped (Ouyang *et al.* 2014). *QPm.lfl-7A* was physically located between the two markers *Xwmc525* and *Xcdo347*. The QTL was 12 Mbp distal to the marker *Xwmc525* and 1 Mbp proximal to the marker *Xcdo347*. The latter is linked to the gene *Pm1* that is associated with the *Lr20/Sr15/Rlnn1* multi-resistance locus (Jayatilake *et al.* 2013; Ouyang *et al.* 2014). Based on the physical localization, it is likely that *QPm.lfl-7A* represents the *Pm1* gene. The *QPm.lfl-6B* peak marker was located 6 Mbp distal from the marker *Xbarc134*, which is linked to the race-specific resistance gene *Pm54* (Hao *et al.* 2015). The gene was recently described in the common winter wheat variety ‘AGS 2000’ as an all-stage resistance gene (Hao *et al.* 2015). Besides the *PmG3M* gene which, however, originated from *T. dicoccoides* (Xie *et al.* 2012), no additional PM genes are known in this region. Therefore, it is likely that the QTL represents the *Pm54* gene, particularly as *QPm.lfl-6B* was also mapped at the seedling stage (Stadlmeier *et al.* 2018).

The STB QTL *QStb.lfl-2B.3* on the long arm of chromosome 2B was mapped to the same genetic region as the *Stb9* gene. The QTL was physically mapped 61 Mbp proximal to the marker *Xwmc317* that is linked to the *Stb9* gene (Chartrain *et al.* 2009). This gene was reported to be active at the seedling stage. As we selected our isolates regarding the absence of seedling resistance, *QStb.lfl-2B.3* is assumed to represent an adult plant stage resistance locus and not *Stb9*. Our QTL was located 14 Mbp distal and 29 Mpb proximal to two other QTL described in literature for field resistance to STB (Eriksen *et al.* 2003; Miedaner *et al.* 2012). The proximal QTL was shown to be active only at the adult plant stage. Therefore, it appears that some way proximal of the seedling resistance gene *Stb9*, an adult plant stage resistance locus exists.

On the short arm of chromosome 2B, *QTs.lfl-2B* and the toxin insensitive locus *Tsc2* were located in the distal region (Faris *et al.* 2013). However, *tsc2* does not segregate in the population, as all founders of BMWpop are insensitive to ToxB (data not shown). However, it has been suggested that adjacent to *Tsc2*, another gene conferring resistance to several races of *P. tritici-repentis* might be present (Li *et al.* 2011). Based on the physical map positions, we conclude that this novel gene is located about 100 Mbp proximal to *Tsc2*. The qualitative TS resistance gene *Tsr3* on the short arm of chromosome 3D is located distal to the marker *Xbarc42* (Tadesse *et al.* 2007). *QTs.lfl-3D* was mapped 300 Mbp distal to this marker. Although the physical distance is large, it is still conceivable that the QTL corresponds to *Tsr3* as *Xbarc42* is not closely linked to that gene (Tadesse *et al.* 2007) and the genetic region is considered to possess a very low recombination rate. The ToxC effector insensitivity locus *Tsc1* is located very distal on the short arm of chromosome 1A (Faris *et al.* 2013), where *QTs.lfl-1A* was also mapped. A comparison based on physical position was not possible due to the lack of publically available sequences for the markers linked to *Tsc1*. However, it could be viewed as unlikely that *QTs.lfl-1A* corresponds to *Tsc1* as the QTL perfectly overlaps with *QStb.lfl-1A* and it has not been reported yet that *Z. tritici* produces the necrotrophic toxin ToxC.

Würschum *et al.* (2017) reported that almost all markers that have been significantly associated with the reduced-height gene *Rht24* have been located in the physical region between 400 and 450 Mbp on chromosome 6A. Our PH QTL on chromosome 6A was mapped to 452 Mbp, indicating that *QHt.lfl-6A* corresponds to *Rht24*.

The frost-tolerance loci *Fr-A1* and *Fr-A2* span a distance of 74 Mbp on chromosome 5A (McIntosh *et al.* 2013). The EET QTL *QEet.lfl-5A* was located in the center of this interval. Furthermore, only 1 Mbp distal to *Fr-A1* the vernalization locus *Vrn-A1* is located. Although functional markers showed that the BMWpop is monomorphic for the *Vrn-A1* allele, the *Vrn-A1* locus can still be considered as a candidate locus for *QEet.lfl-5A*. It was shown that copy number variation of *Vrn-A1* controls flowering time in winter varieties (Díaz *et al.* 2012). Therefore, it is likely that *QEet.lfl-5A* represents one of these three loci. On chromosome 5B, *QEet.lfl-5B* was located in the same genetic region as the vernalization gene *Vrn-B1* but 115 Mbp distal (Yan *et al.* 2003). However, winter wheat varieties normally carry homozygote recessive ‘winter’ alleles at the *Vrn* loci (Stelmakh 1987; Watson-Haigh *et al.* 2018). The closely linked flowering pathway gene *PHYTOCHROME C* (*PHYC*) has been shown via studies of artificial mutants in the tetraploid wheat *T. durum* to play a role in the control of flowering time (Chen *et al.* 2014). Therefore, it is uncertain whether *QEet.lfl-5B* corresponds to the *Vrn-B1* gene, or to another linked locus such as *PHYC*.

On chromosome 2B, three QTL for STB were mapped. However, *QStb.lfl-2B.1* and *QStb.lfl-2B.2* appear to represent resistance loci conditioned by leaf morphology because both overlapped with QTL for LAD. A relationship of LAD and STB could be expected since spray inoculation took place from the top of the canopy. It is already known that morphological traits such as PH and EET can be linked to STB resistance QTL (Eriksen *et al.* 2003). Besides the two aforementioned QTL, no further cQTL between disease and agro-morphological traits were detected, despite the fact that PH and EET were significantly correlated with STB and TS. However, we can assume that *QStb.lfl-2B.1* represents a resistance locus mainly conditioned, beside the leaf morphology, by heading date, as the peak marker is a functional marker for the major photoperiod response gene, *Ppd-B1*. Although this locus was segregating in the population, it was not possible to detect a QTL for EET in this region in the analysis across TEs. *Ppd-B1* was only mapped in the TE 16FS1 as a QTL for EET. This may be explained by the significant genotype * environment interaction.

Two genetic regions on the short arm of chromosome 1A (1AS) and the long arm of chromosome 7A (7AL) were identified to be associated with all three diseases. On chromosome 1A, the QTL for STB and TS showed a perfect overlap and the PM QTL was about 5 Mbp distally located. Based on the additive effects of the founders, a higher level of resistance to PM came along with a higher level of resistance to both necrotrophic diseases. On chromosome 7AL, the peak marker for *QPm.lfl-7A* was mapped 14 Mbp distal to *QTs.lfl-7A.2*. In the analysis across TEs, no QTL for STB was detected. However, in the TE 16FS1 a QTL for STB was mapped 5 Mbp distal to *QPm.lfl-7A*. While the founder effects for PM and STB showed a similar trend, the effects for TS were antagonistic. This implies that the genetic region on chromosome 7AL simultaneously confers resistance to PM and STB and susceptibility to TS.

Recombinant inbred lines, which are selfed to around the F_5_ generation, are valuable resources for developing heterogeneous inbred families (HIFs; Tuinstra *et al.* 1997). HIFs are characterized by a high identical genetic background combined with a different genetic constitution at the genomic region of interest. Pairs of progenies of such a family, which carry contrasting alleles at a target QTL, serve as a tool for an accurate investigation of the effects of these alleles. Additionally, crossing such pairs of progeny together in order to generate genetic recombination within the target QTL interval can be used to narrow the QTL target region to resolutions that may enable identification of the underlying gene and genetic variant. Accordingly, the future development of HIFs for QTL identified in this study will allow the precise phenotypic and molecular investigations of Mendalized QTL to be undertaken.

In summary, we validated the BMWpop MAGIC population as valuable plant genetic resource to investigate the genetic relations between various traits within a single population. We identified two genetic regions on chromosome 1AS and 7AL, which are associated with all three diseases. We conclude that parallel breeding for resistance to PM, STB, and TS appears feasible, although specific genetic regions such as on chromosomes and 7AL with contrasting effects for the different diseases should be kept in mind.
